# Human Genetics in Rheumatoid Arthritis Guides a High-Throughput Drug Screen of the CD40 Signaling Pathway

**DOI:** 10.1371/journal.pgen.1003487

**Published:** 2013-05-16

**Authors:** Gang Li, Dorothée Diogo, Di Wu, Jim Spoonamore, Vlado Dancik, Lude Franke, Fina Kurreeman, Elizabeth J. Rossin, Grant Duclos, Cathy Hartland, Xuezhong Zhou, Kejie Li, Jun Liu, Philip L. De Jager, Katherine A. Siminovitch, Alexandra Zhernakova, Soumya Raychaudhuri, John Bowes, Steve Eyre, Leonid Padyukov, Peter K. Gregersen, Jane Worthington, Rheumatoid Arthritis Consortium International (RACI), Namrata Gupta, Paul A. Clemons, Eli Stahl, Nicola Tolliday, Robert M. Plenge

**Affiliations:** 1 Division of Rheumatology, Immunology, and Allergy and Division of Genetics, Brigham and Women's Hospital, Harvard Medical School, Boston, Massachusetts, United States of America; 2 Medical and Population Genetics Program, Chemical Biology Program, Broad Institute, Cambridge, Massachusetts, United States of America; 3 Department of Statistics, Harvard University, Cambridge, Massachusetts, United States of America; 4 Chemical Biology Platform, Broad Institute, Cambridge, Massachusetts, United States of America; 5 Chemical Biology Program, Broad Institute, Cambridge, Massachusetts, United States of America; 6 Department of Genetics, University Medical Center Groningen and University of Groningen, Groningen, The Netherlands; 7 Biological and Biomedical Sciences Program, Health Sciences and Technology Program, Harvard Medical School, Boston, Massachusetts, United States of America; 8 Analytical and Translational Genetics Unit, Massachusetts General Hospital, Boston, Massachusetts, United States of America; 9 School of Computer and Information Technology, Beijing Jiaotong University, Beijing, China; 10 Program in Translational NeuroPsychiatric Genomics, Institute for the Neurosciences Department of Neurology, Brigham and Women's Hospital, Boston, Massachusetts, United States of America; 11 Department of Medicine, University of Toronto, Toronto, Ontario, Canada; 12 Mount Sinai Hospital, Samuel Lunenfeld Research Institute and Toronto General Research Institute, Toronto, Ontario, Canada; 13 Department of Rheumatology, Leiden University Medical Centre, Leiden, The Netherlands; 14 Arthritis Research UK Epidemiology Unit, Musculoskeletal Research Group, University of Manchester, Manchester Academic Health Sciences Centre, Manchester, United Kingdom; 15 NIHR Manchester Musculoskeletal Biomedical Research Unit, Central Manchester NHS Foundation Trust, Manchester Academic Health Sciences Centre, Manchester, United Kingdom; 16 Rheumatology Unit, Department of Medicine, Karolinska Institutet and Karolinska University Hospital Solna, Stockholm, Sweden; 17 The Feinstein Institute for Medical Research, North Shore–Long Island Jewish Health System, Manhasset, New York, United States of America; University of Oxford, United Kingdom

## Abstract

Although genetic and non-genetic studies in mouse and human implicate the CD40 pathway in rheumatoid arthritis (RA), there are no approved drugs that inhibit CD40 signaling for clinical care in RA or any other disease. Here, we sought to understand the biological consequences of a *CD40* risk variant in RA discovered by a previous genome-wide association study (GWAS) and to perform a high-throughput drug screen for modulators of CD40 signaling based on human genetic findings. First, we fine-map the *CD40* risk locus in 7,222 seropositive RA patients and 15,870 controls, together with deep sequencing of *CD40* coding exons in 500 RA cases and 650 controls, to identify a single SNP that explains the entire signal of association (rs4810485, *P* = 1.4×10^−9^). Second, we demonstrate that subjects homozygous for the RA risk allele have ∼33% more CD40 on the surface of primary human CD19+ B lymphocytes than subjects homozygous for the non-risk allele (*P* = 10^−9^), a finding corroborated by expression quantitative trait loci (eQTL) analysis in peripheral blood mononuclear cells from 1,469 healthy control individuals. Third, we use retroviral shRNA infection to perturb the amount of CD40 on the surface of a human B lymphocyte cell line (BL2) and observe a direct correlation between amount of CD40 protein and phosphorylation of RelA (p65), a subunit of the NF-κB transcription factor. Finally, we develop a high-throughput NF-κB luciferase reporter assay in BL2 cells activated with trimerized CD40 ligand (tCD40L) and conduct an HTS of 1,982 chemical compounds and FDA–approved drugs. After a series of counter-screens and testing in primary human CD19+ B cells, we identify 2 novel chemical inhibitors not previously implicated in inflammation or CD40-mediated NF-κB signaling. Our study demonstrates proof-of-concept that human genetics can be used to guide the development of phenotype-based, high-throughput small-molecule screens to identify potential novel therapies in complex traits such as RA.

## Author Summary

A current challenge in human genetics is to follow-up “hits” from genome-wide association studies (GWAS) to guide drug discovery for complex traits. Previously, we identified a common variant in the *CD40* locus as associated with risk of rheumatoid arthritis (RA). Here, we fine-map the *CD40* signal of association through a combination of dense genotyping and exonic sequencing in large patient collections. Further, we demonstrate that the RA risk allele is a gain-of-function allele that increases the amount of CD40 on the surface of primary human B lymphocyte cells from healthy control individuals. Based on these observations, we develop a high-throughput assay to recapitulate the biology of the RA risk allele in a system suitable for a small molecule drug screen. After a series of primary screens and counter screens, we identify small molecules that inhibit CD40-mediated NF-kB signaling in human B cells. While this is only the first step towards a more comprehensive effort to identify CD40-specific inhibitors that may be used to treat RA, our study demonstrates a successful strategy to progress from a GWAS to a drug screen for complex traits such as RA.

## Introduction

Rheumatoid arthritis (RA) is a common autoimmune disease for which there is no known cure. A diverse number of biological pathways are altered in patients with RA, which impinge on a wide-variety of cell types, tissue types and organ systems – innate immune cells (e.g., neutrophils, dendritic cells, mast cells, platelets), adaptive immune cells (e.g., B and T cells), bone, cartilage, synovial fibroblasts, vascular cells, brain, muscle, and fat [1]. Accordingly, the task of sorting through which biological pathways cause disease, as compared to those pathways that are simply a consequence of disease, is a daunting challenge. Without knowing the critical causal pathways, it is very difficult to develop novel therapeutics to treat or cure RA.

There are fundamental principles of human genetics that make it a promising strategy to identify critical biological pathways and novel therapeutic targets in complex traits such as RA [2]. Since risk alleles are randomly assigned at meiosis, are independent of non-genetic confounding, and are unmodified by the disease itself, human genetics can help distinguish between cause and consequence. Moreover, risk alleles indicate if a pathway is up or down regulated in disease – a critical first step in drug development. Risk alleles help calibrate the amount of target modulation that is tolerable in humans, as gain-of-function and loss-of-function mutations in the same gene can be assessed for clinical phenotypes in carriers of these mutations. Consistent with these concepts, known drug targets that are safe and effective in humans appear on the list of genes identified by genome-wide association studies (GWAS) of common diseases [3], which suggests that other GWAS hits represent targets worthy of further investigation [4].

However, there are important challenges in translating SNP associations from human genetics (and GWAS in particular) to novel therapeutics. First, the causal gene must be identified within the risk locus, as there are often multiple genes in the region of linkage disequilibrium. Compounding this challenge, most GWAS hits are to non-coding variants that cannot pinpoint specific genes. Second, the risk allele must be experimentally validated as gain- or loss-of-function in a relevant human tissue, in order to guide whether a drug should inhibit or activate (respectively) the target of interest. Third, the biology of the risk allele should be recapitulated in an assay system suitable for a high-throughput screen (HTS). And fourth, the HTS should demonstrate performance characteristics that make it robust for screening large chemical libraries.

The CD40-CD40L pathway represents a good example of a pathway for which human genetics may help guide drug development. The pathway is upregulated in multiple diseases [5]–[7], including autoimmune diseases such as RA [8]–[15]. GWAS identified a common variant in the *CD40* locus that increases risk of RA, which suggests that CD40 upregulation is a cause rather than a consequence of chronic inflammation [16]. Loss-of-function mutations in both *CD40* and *CD40L* result in immunodeficiency, but only in the homozygous state, indicating that 50% inhibition of CD40-CD40L signaling (as observed in heterozygous mutation carriers) should be safely tolerated in humans [17].

Despite these findings, there are currently no approved drugs that inhibit CD40-CD40L signaling, and there are no drugs in clinical trials (www.clinicaltrials.gov). Others have developed small molecules that disrupt CD40-CD40L binding, but these compounds have not been tested in humans [18]. Antibodies to CD40L were effective in treating inflammatory diseases, but resulted in thrombotic events due to the presence of CD40L on platelets [19]–[28]. Human genetics suggests that inhibiting intracellular signaling of CD40-mediated signaling will also likely be effective in humans, without adverse events related to thrombosis.

Here, we demonstrate a strategy that uses findings from GWAS to guide the development of a drug screen for the identification of small molecules inhibiting the CD40-CD40L intracellular signaling pathway. We hypothesize that such molecules might in turn be safe and effective in treating inflammation observed in RA patients. We set out (1) to investigate the biology of the *CD40* risk allele, by fine-mapping the *CD40* locus and analyzing the function of the risk allele in primary B cells from healthy donors; (2) to recapitulate the biology of the *CD40* risk allele in an assay system suitable for a high-throughput screen (HTS) of small molecule drugs; and (3) to conduct a pilot HTS to search for known and novel inhibitors of CD40-mediated signaling in human B cells.

## Results

### The RA risk allele at *CD40* locus induces an increase of CD40 protein level on the surface of B cells

**Figure 1:**
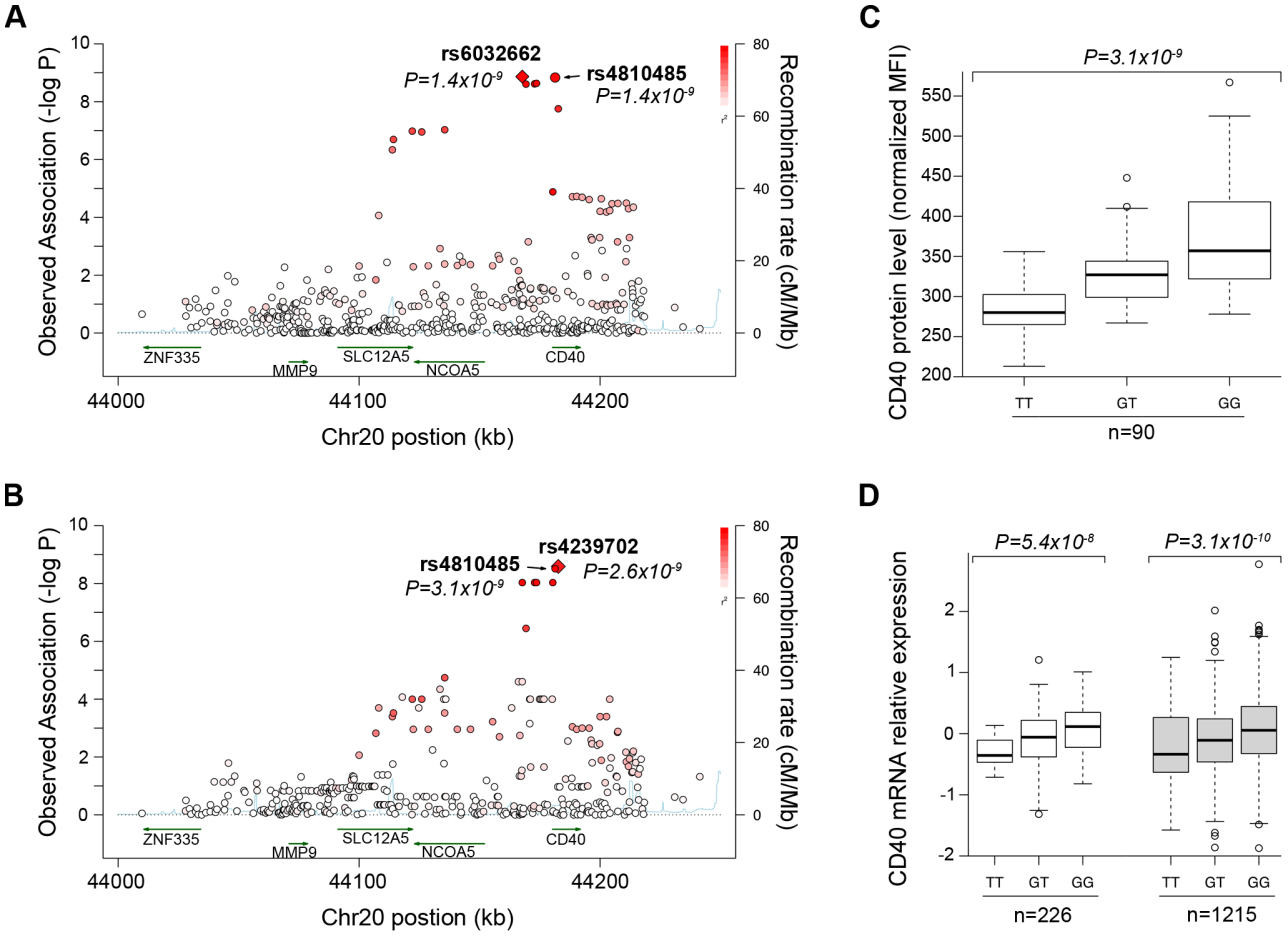
Genetic data on risk of RA and CD40 protein levels. (A) The regional association plot from analysis of Immunochip (iChip) data in 7,222 CCP+ cases and 15,870 controls. Gene location is shown along the bottom of the graph, with observed –log(P) value along the left Y-axis and recombination rate along the right Y-axis. Each SNP is plotted is a circle, with color scheme (red to white) in reference to the extent of linkage disequilibrium with the index SNP, rs4810485 (labeled as a diamond). (B) The regional association plot from analysis of iChip data and CD40 protein levels in 90 healthy control individuals. (C) A box-whisker's plot of SNP (rs4810485) and CD40 protein levels in B cells from healthy control individuals, where T = non-risk allele and G = risk allele. (D) A box-whisker's plot of SNP (rs4810485) and *CD40* mRNA levels in PBMC's from two separate collections (total of 1,441 healthy control individuals); T = non-risk allele and G = risk allele.

We first performed comprehensive genotyping at the *CD40* risk locus to fine-map the causal allele. Additional details can be found in Text S1. We used a dataset in which RA case-control samples were genotyped at high density across the *CD40* locus with the Illumina Immunochip platform (Eyre et al. Nat Genet. in press). In total, we analyzed 492 SNPs in 7,222 seropositive RA patients and 15,870 controls (Table S1). As shown in Figure 1A, our analysis revealed that the strongest signal of association was shared by 2 “equivalent” non-coding SNPs in linkage disequilibrium (LD) at r^2^ = 0.98, rs6032662 and rs4810485 (*P* = 1.4×10^−9^, OR = 1.17 per copy of the risk allele). After conditional analysis, no additional signal of association remained, indicating that one of these 2 SNPs, or one of the other six equivalent non-coding SNPs in LD at r^2^>0.80, is the underlying causal allele at this locus (Figure S1A, Table S2A). We refer to rs4810485 as the index SNP; the major G allele is the RA risk allele and the minor T allele is the non-risk allele.

A previous study showed that a perfect proxy of the *CD40* RA risk allele increases CD40 protein on the surface of B lymphocyte cells from 23 healthy individuals [29]. To confirm and extend these findings, we developed a flow cytometry assay to measure CD40 on the surface of primary human CD19+ B cells. We demonstrated high reproducibility of the assay on blood samples drawn from the same individual >3 months apart (r^2^ = 0.76, Figure S2). We measured CD40 protein levels from 90 healthy control subjects. We performed high-density SNP genotyping across the *CD40* locus, using the same genotyping array as in our case-control study of RA risk. Strikingly, the strongest signal of association across the *CD40* locus was at the RA risk allele (Figure 1B; *P* = 3×10^−9^, Table S2). After conditional analysis, no additional SNP was significant (Figure S1B). Healthy control subjects homozygous for RA risk allele have ∼33% more CD40 on the surface of primary human CD19+ B lymphocytes than subjects homozygous for the non-risk allele (Figure 1C). The RA risk allele (the G allele of rs4810485) explains 31% of variation observed in CD40 protein level in these healthy control subjects, and was the strongest signal among the genome-wide set of SNPs tested for association with CD40 protein levels (Figure S1C).

To complement this finding, we examined *CD40* gene expression in peripheral blood mononuclear cells of 1,469 unrelated individuals [30]. As shown in Figure 1D, we found that the RA risk allele was an expression quantitative trait locus (eQTL) on *CD40* gene expression (*P* = 8.2×10^−13^). Similar findings have been reported for the RA risk allele in other immune cell types [31], [32].

Taken together, our data demonstrate unequivocally that the RA risk allele (the G allele of rs4810485) is a gain-of-function mutation that leads to increased level of CD40 on the surface of primary human CD19+ B cells (and possibly other immune lineages within PBMC's).

### CD40 influences p65 phosphorylation in a B-cell line (BL2)

**Figure 2:**
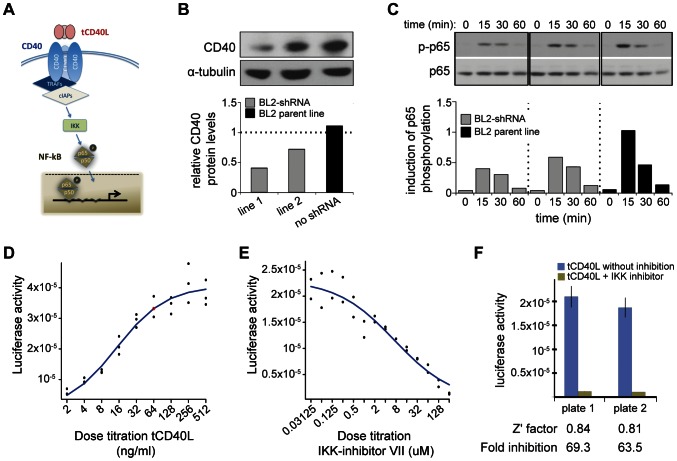
CD40 knockdown and CD40-luciferase assay in BL2 cells. (A) Schematic of the canonical CD40 – NF-|B signaling pathway in B cells. (B) RNAi perturbation of *CD40* in two distinct clones derived from BL2 cells decreases CD40 protein levels by 55% (left) and 40% (middle) compared to the BL2 parent line (black, right); (C) More CD40 on the surface of BL2 cells increases RelA (p65) phosphorylation following activation with tCD40L, as measured by Western blot, with maximum activation at 15 minutes. Results are shown for the same two shRNA lines and parental BL2 cell line as in (B). This is a representative example of multiple experiments. (D) Titration of tCD40L leads to increased luciferase activity. Each experiment was performed in triplicate. The red circle represents ∼80% maximum luciferase activity (64 ng/ml tCD40L). Luciferase activity at baseline (i.e., no tCD40L activation) was subtracted from each measurement to plot results. (E) Titration of IKK inhibitor VII leads to inhibition of luciferase activity following tCD40L activation. Each experiment was performed in duplicate. (F) The luciferase assay is robust, with Z'-factor>0.80 and >60-fold inhibition of luciferase activity without killing cells across different plates.

We next sought to determine the biological consequences of having more CD40 on the surface of B cells, in order to determine the most appropriate assay for a drug screen. Engagement of CD40 by its trimerized ligand (tCD40L) leads to phosphorylation of p65, a subunit of NF-κB (Figure 2A). To determine the effect of p65 phosphorylation in a human B cell line (BL2) with varying levels of CD40 protein, we derived clones in which *CD40* mRNA was knocked-down with shRNA [33]. In two independent cell lines, we observed CD40 protein levels at 40% and 55% compared to the BL2 parent line, respectively (Figure 2B). We used the parent BL2 line and two BL2/shRNA lines to activate the CD40 signaling pathway with tCD40L. In both BL2/shRNA cell lines, we observed a reproducible decrease in phosphorylation of p65 at Ser536 at 15 and 30 minutes following tCD40L activation. The levels of NF-κB p65 phosphorylation, as measured by Western blot, correlated with the levels of CD40 protein across all three B cell lines (Figure 2C). That is, more CD40 on the surface of B cells (as is the case for carriers of the RA risk allele) has increased activation of the classical NF-κB pathway (as measured by phosphorylation of NF-κB p65).

### HTS to identify inhibitors of CD40-mediated NF-κB signaling

A Western blot is not suitable for a high-throughput screen (HTS). Based on our functional analysis, we developed a luciferase reporter assay that can be used in an HTS to identify inhibitors of CD40 signaling pathway. For this assay, we generated a BL2 cell line (BL2-NFκB-Luc) that was transfected with a luciferase reporter construct driven by a basal promoter element (TATA box) joined to tandem repeats of the NF-κB response element. To optimize conditions for an HTS, we performed a series of experiments with BL2-NFκB-Luc cells. First, we performed a titration of tCD40L, and found approximately 80% activation at 64 ng/ml tCD40L (Figure 2D). Second, we determined the optimal time course following 64 ng/ml tCD40L activation, and found maximum activation (5.6-fold induction) at 4.5 hours. Third, we performed a titration of a known inhibitor of the classical NF-κB signaling pathway, IKK inhibitor VII (Milipore) (Figure 2E). To confirm that the decrease of NF-κB activity by this inhibitor is not due to cytotoxicity, we used an anti-PARP antibody (116-kDa poly-ADP-ribose nuclear polymerase) to demonstrate by Western blot that the decrease in NF-κB phosphorylation following IKK inhibition was not simply due to cell death (Figure S3). And fourth, we determined the fold-increase and fold-inhibition of luciferase activity following tCD40L activation and IKK inhibition, respectively. We observed robust performance of our assay under a specific set of conditions (Figure 2F), with a Z'-factor≈0.8 [34] (where a Z'-factor of >0.50 is considered appropriate for a small-molecule screen [35]).

**Figure 3:**
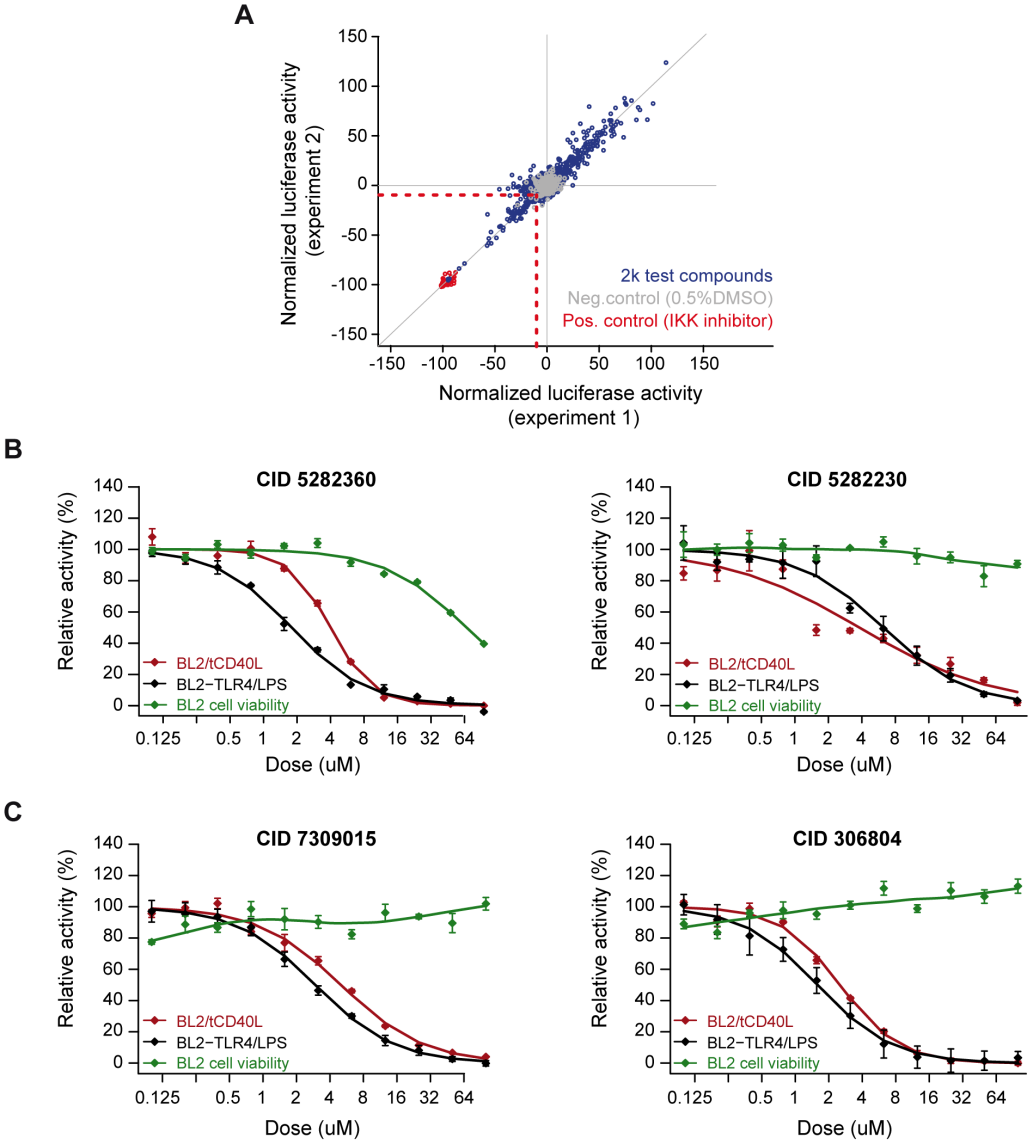
Small molecule screen of CD40-mediated NF-kB signaling in BL2 cells. (A) Results from duplicate experiments screening 1,982 compounds. Red circles are our positive control (IKK inhibitor VII); grey circles are our neutral controls (DMSO only); and blue circles are test compounds. The red dashed line indicates >2SD from the mean of the neutral controls, which defines our “hit” compounds (n = 81 compounds). (B) Dose-response curves for two compounds known to inhibit inflammation [CID = 5282230 (tranilast)] or NF-|B signaling [CID = 5282360 (4-hydroxy-estradiol)] in the BL2-NF|B-Luc cell lines. (C) Dose-response curves for two compounds not previously implicated in inflammation, NF-κB signaling, CD40 signaling, or other biological pathways related to rheumatoid arthritis: CID = 306804, [4-(1-acetyl-4-oxo-2H-3,1-benzoxazin-2-yl)phenyl] acetate; and CID = 7309015, 8-[(Z)-3-(3,4-dimethoxyphenyl)prop-2-enoyl]-7-hydroxy-4-methylchromen-2-one. Red line = cells activated with tCD40L; black line = cells activated with either CD40 or LPS (in BL2-TLR4-NFκB-Luc cells); green line = cell toxicity, as measured by CellTiter-Glo.

We optimized our luciferase assay in a 384-well format. We conducted a pilot screen of 2,240 chemical compounds (of which 1,982 are in PubChem), each assayed in duplicate experiments. The chemical compounds comprise bioactive compounds (including FDA-approved drugs), commercially available drug-like molecules, targeted collections (e.g., biased for kinases), stereochemically-diverse compounds, and purified natural products. Following normalization of luciferase activity to correct for variability across plates, we determined fold-change in luciferase activity relative to our positive (IKK inhibitor VII) and neutral (0.5% DMSO) controls. The Z'-factors for this pilot screen ranged from 0.63–0.85 (average of 0.79). We observed strong correlation between the two experimental replicates (r^2^ = 0.94; Figure 3A).

We identified 81 compounds (4.1% of all compounds) with >2SD decrease in luciferase activity relative to DMSO controls (which we refer to as preliminary “hit” compounds; Table S3). Even at this liberal threshold, there are more compounds that decrease luciferase activity than would be expected by chance alone (*P* = 0.006). As further evidence that many of these hit compounds represent true positive findings, we observed enrichment of known anti-inflammatory agents using text-mining of PubChem annotations [36], [37] (Figure S5A–S5C) and enrichment of shared chemical structure pertaining to corticosteroids and their analogs (Figure S6 and Table S5), which are known NF-κB inhibitors [38].

### Confirmatory screens, counter-screens, and testing compounds in primary CD19+ B cells

To confirm inhibition of CD40-mediated NF-κB signaling, we conducted a series of counter-screens using our primary assay (tCD40L-activated BL2-NFκB-Luc cells) and another B cell line, Ramos RA-1, transfected with the same NF-κB luciferase reporter construct (Ramos-NFκB-Luc). In addition to activation with tCD40L, we activated BL2-NFκB-Luc cells with LPS (which binds to TLR4 and signals through the classical NF-κB pathway) and Ramos-NFκB-Luc cells with TNF-alpha (which also signals through the classical NF-κB pathway). We measured luciferase activity following exposure with each of the 73 compounds across 8 different concentrations (stock concentrations were not available for 8 compounds). For 20 of the 73 compounds (∼1% of all compounds tested), we observed consistent, dose-dependent inhibition across all four assays (BL2-NFκB-Luc cells activated with tCD40 and LPS; Ramos-NFκB-Luc activated with tCD40L and TNF-alpha), without evidence of cellular toxicity (Table S6).

To ensure that inhibition was due to effect of the chemical compound and not an experimental artifact, we re-ordered 14 of the compounds that were available commercially, confirmed chemical structure and purity using high performance liquid chromatography and mass spectrometry, and re-tested these compounds using the same BL2- and Ramos-NFκB-Luc assays. We confirmed that several compounds known to inhibit inflammation (e.g., indoprofen; PubChem Compound ID [CID] 3718) [39] or NF-κB signaling (e.g., 3-[(4-methoxyphenoxy)methyl]benzohydrazide; CID 843208) [40] are potent inhibitors in our assays. We also identified corticosteroids (e.g., 4-hydroxy-estradiol; CID 5282360) and inhibitors of inflammatory arthritis in murine models of RA (tranilast; CID 5282230) [41], [42]. Representative examples are shown in Figure 3B and Figure S7.

**Figure 4:**
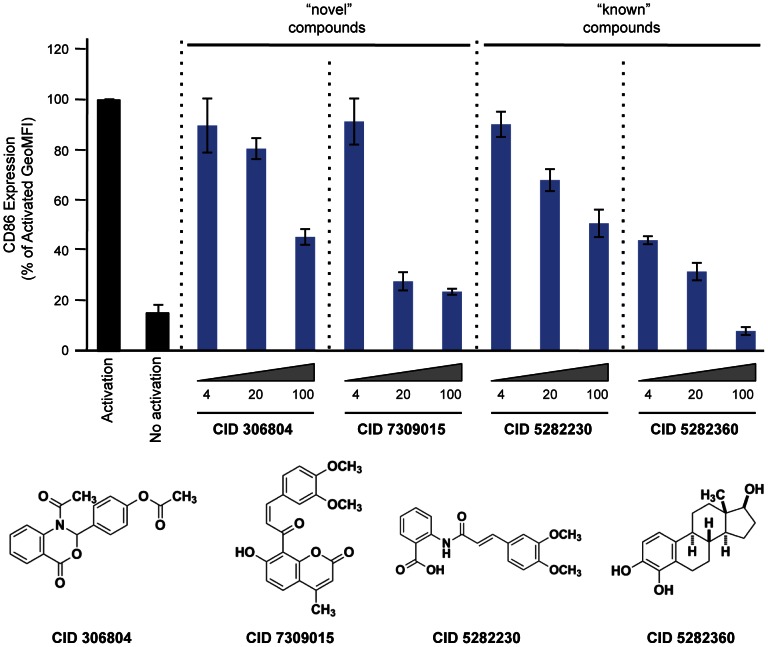
Effect of compounds on CD86 expression in primary CD19+ B cells. Purified human CD19+ primary B cells were incubated with 10 ng/ml IL4 alone (“No activation”) or IL4+64 ng/ml tCD40L (“Activation”), together with different concentrations of drugs for 48 hours. CD86 expression was measured by PE GeoMFI on CD19+ gated B cells. The chemical structure of each compound is shown.

Equally importantly, however, we identified 2 chemical compounds not previously implicated in inflammation, NF-κB signaling or inflammatory arthritis (Figure 3C and Figure 4). For both, the relative IC_50_ was <20 µM (Table 1), with >50% decrease in luciferase activity (Table 2).

**Table 1:** Relative IC_50_ for two “known” and two “novel” compounds in both BL2-NFκB-Luc and Ramos-NFκB-Luc cell lines.

	BL2/tCD40L	BL2-TLR4/LPS	Ramos/tCD40L	Ramos/TNFa
**“known” compounds**				
5282230	3.8	6.3	7.1	8.1
5282360	4.1	1.8	2.1	1.3
**“novel” compounds**				
306804	2.5	1.6	9.4	6.7
7309015	5.0	3.0	18.3	16.4

Relative IC_50_ is the concentration required to bring the dose-response curve to the halfway point between the top and bottom plateaus of the curve.

**Table 2:** Percent maximum inhibition for two “known” and two “novel” compounds in both BL2-NFκB-Luc and Ramos-NFκB-Luc cell lines.

	BL2/tCD40L	BL2-TLR4/LPS	Ramos/tCD40L	Ramos/TNFa
**“known” compounds**				
5282230	54.7%	73.7%	86.4%	87.4%
5282360	87.0%	54.1%	95.1%	92.7%
**“novel” compounds**				
306804	83.8%	51.8%	91.7%	90.5%
7309015	90.2%	63.3%	78.3%	85.7%

For each compound, we calculate the maximum amount of inhibition observed at the highest concentration of drug relative to zero luciferase activity.

Finally, we tested 2 “known” and 2 “novel” compounds for their ability to inhibit tCD40L-mediated NF-κB signaling in primary CD19+ B cells from healthy control subjects. We used flow cytometry to measure CD86 cell-surface protein levels, as CD86 expression is up-regulated upon activation of B cells with CD40L [43]. We observed dose-dependent decrease in CD86 expression with all 4 compounds (Figure 4). Thus, we confirmed that the 2 known inhibitors and the 2 novel compounds identified in our HTS inhibit the NF-κB signaling pathway in primary CD19+ B cells from healthy donors.

## Discussion

Our study exploits fundamental principles of human genetics to guide a small molecule drug screen in RA. We show that upregulation of the CD40-CD40L signaling pathway is a cause rather than a consequence of disease, as the RA risk allele increases levels of CD40 on the surface of B lymphocyte cells and increases CD40-mediated NF-kB signaling. Based on our genetic findings, a prediction is that drugs that attenuate CD40-mediated NF-kB signaling will either protect from RA or treat symptoms in patients with active disease. From our screen, we identify two compounds that support this hypothesis: tranilast, which reduces inflammation in a mouse model of RA [41], and a corticosteroid, which is a potent anti-inflammatory drug used to treat RA [44], [45]. Equally important, we discover 2 novel small-molecules that inhibit CD40 signaling through the classical NF-κB pathway in primary CD19+ B cells.

There are few examples where GWAS was used to guide drug discovery. One example is *PCSK9*, where a loss-of-function variant is associated with lower levels of LDL cholesterol and protection from cardiovascular disease [46]–[49]. However, the original finding implicating *PCSK9* and LDL cholesterol came not from GWAS, but from sequencing in families with autosomal dominant high LDL levels and an increased incidence of coronary heart disease [50]. In 2012, a randomized control trial was published that a monoclonal antibody to PCSK9 significantly reduced LDL cholesterol levels in healthy volunteers and in subjects with hypercholesterolemia [51], [52]. Another example is *BCL11A* and persistence of fetal hemoglobin in sickle cell anemia. In 2008, a GWAS found an association with a common, non-coding variant of the hemoglobin silencing factor gene, *BCL11A*, and HbF expression [53], [54]. Based on these data, together with data from animal models [55], repressors of BCL11A are under development for the treatment of sickle cell disease [56].

Our study illustrates another example, as we demonstrate that genetic findings can be instrumental in developing optimal high-throughput drug screens. In RA, many biological pathways have been implicated. Consequently, identifying relevant pathways is critical for the development of molecules that will be effective in treating the disease. Our strategy successfully links an RA risk allele to a biological process suitable for an HTS. First, we show unequivocally that the RA risk allele leads to increased levels of CD40 protein on the surface of CD19+ B cells, thereby establishing a causal link between increased CD40 protein levels and risk of RA. Second, we establish a direct relationship between amount of CD40 on the surface of B cells and an intracellular biological pathway, NF-kB signaling. In doing so, we recapitulate the effect of the CD40 risk allele in an assay system suitable for an HTS.

There are important limitations of our study. First, the chemical library tested in our study is small relative to libraries in academic centers and industry (which often contain hundreds of thousands to millions of compounds) [57], [58]. Second, our screen did not identify inhibitors specific to CD40 signaling. Whether a more selective CD40 inhibitor would be a better therapeutic than a more general inhibitor requires additional studies. That our HTS identified two “known” drugs that inhibit inflammation reinforces that our general strategy is successful. Third, we have not yet tested our compounds in animal models of RA. However, one of our known compounds, tranilast, has been shown by others to inhibit collagen-induced arthritis in the mouse [41]. Fourth, we do not yet know the target of our “novel” small molecule inhibitors of CD40-mediated NF-κB signaling. One of these compounds (CID 7309015) has been annotated in PubChem as an inhibitor of retinoic acid-related orphan receptor (ROR) gamma, a transcription factor that has a central role in the differentiation of CD4+ Th17 cells. However, this PubChem annotation has not yet been linked to a PubMed manuscript. The other compound has not been confirmed as active in any PubChem assay, and therefore represents a novel tool compound for further study.

In conclusion, we demonstrate a strategy to translate GWAS findings into HTS to identify novel small-molecule inhibitors of the CD40 signaling pathway. Given the wealth of GWAS data that has accumulated in recent years, human genetics represents a promising approach to develop safe and effective therapies to treat complex human diseases such as RA.

## Materials and Methods

Our study was approved by the institutional review board (IRB) at our institutions.

### Genetic analysis of CD40 risk locus in RA case-control samples

Six case-control collections were included for genotyping using the Illumina Immunochip platform (Table S1), as part of the RACI consortium [59]; the GWAS datasets have been previously described, and include 4 collections that did not overlap with the Immunochip dataset [60]. All six Immunochip datasets were clustered together using the Illumina Genome Studio algorithm. Initial data filtering steps in GenomeStudio included: removal of samples with call rate<90% and removal of SNPs with poor clustering quality metrics (call frequency<0.98, cluster separation<0.4). Further quality control was performed in the six individual population datasets separately. First, samples with call rate <99% were excluded. Second, SNPs with call rate <99% in either the RA cases or controls were excluded. To address population stratification, we selected a set of common SNPs (MAF>5%), pruned to remove SNPs in LD. We calculated pairwise identity-by-state (IBS) statistics using PLINK [61], and removed one individual from each pair of individuals who were 2^nd^ degree or closer relatives. Principal components analysis (PCA) was subsequently performed using EIGENSTRAT [62]. After exclusion of individuals of non-European ancestry, as determined by clustering with CEU HapMap (phase II), a second PCA was performed to further remove outliers. Cases with anti-CCP negative or missing anti-CCP status data were removed, leaving 7,222 CCP+ cases and 15,870 controls for association analysis. To avoid duplicate samples, we used IBS estimates to remove related samples between the Immunochip and GWAS collections. Specifically, we selected a set of genotyped SNPs with missing-genotype rate<0.5%, MAF >5% and Hardy-Weinberg equilibrium (HWE) *P*>10^−3^ that were shared across all 10 collections. When related samples were identified (siblings or duplicates), the sample from the GWAS data was removed (to preferentially keep genotyped data rather than imputed data in the subsequent association analyses), bringing the total sample size to 9,785 seropositive RA cases and 33,742 controls (Table S1). Finally, we computed a chi-square test to assess the difference in missingness between cases and controls and removed SNPs with a *P*_missing_<10^−2^, together with SNPs in departure form Hardy-Weinberg equilibrium (*P*_HWE_>5.7×10^−7^).

To test for association with risk of RA, we used PLINK to conduct logistic regression analysis of the six Immunochip RA case-control status, including 10 eigenvectors as covariates. We conducted an inverse-variance weighted meta-analysis to combine the results across the 6 collections, for the 156,520 SNPs across the genome with results in one or more collections, including 492 SNPs across the *CD40* locus. We also computed Cochran's Q statistic and I^2^ statistic to assess heterogeneity across collections. Meta-analysis and heterogeneity statistics computation was adapted from the MANTEL program.

Sequence data at the *CD40* locus was generated as part of a larger experiment to perform pooled sequencing of 25 RA risk genes (Supplementary Material). Using Syzygy (a pooled variant caller) [63], we estimated the allele frequencies in the overall sample set (500 cases and 650 controls of European ancestry geographically and genetically matched). We observed a strong correlation between genotype frequencies available from our GWAS data and frequencies estimated using the method in Syzygy indicating accurate experimental recovery of the pool composition. For the *CD40* region, we had no coverage of exon 1, but near complete coverage of the remaining eight exons (Figure S4). After stringent quality control (Supplementary Material), we observed 4 coding variants at the *CD40* locus: two coding-synonymous SNPs and two missense SNPs. None of the SNPs was associated with RA either in a single-SNP analysis or in a gene-burden test (Table S2B).

### Measurement and genetic analysis of CD40 levels

CD40 protein levels on the surface of unstimulated CD19^+^ B cells were measured in healthy control subjects from the PhenoGenetic Project of Brigham and Women's Hospital, a living biobank of 1,739 subjects free of chronic inflammatory and infectious diseases recruited from the general population of Boston, MA. Subjects used in this experiment were randomly selected from the biobank. Fresh PBMCs were isolated from 10 ml blood with 5 ml Ficoll-Hypaque (GE; Cat#07908). PBMCs were washed once in 0.1%BSA/PBS and blocked with FcR blocking reagent (Milteyi Biotec; Cat.#120-000-442). After red blood cells were lysed in 10 ml human red blood cell lysis buffer, 0.25×10^6^ isolated PBMCs were double-labeled with an anti-CD19-FITC (eBioscience; Cat.#11-0199-73) and anti-hCD40/TNFRSF5-PE (R&D; Cat.#FAB6321P). The CD40 levels were measured by FACS analysis with PE-GeoMFI on CD19^+^ cells. As a negative control, an anti-IgG2B-PE (R&D; Cat.#IC0041P) was used; in addition, we used frozen BL2 cells to normalize for day-to-day variation. In total, 97 subjects had both CD40 protein levels measured and genotyping generated using the Immunochip beadset at Yale University. The same initial data-filtering steps described above were performed. Following QC, 90 samples with a call rates >99% were included in the analysis. After HapMap phase III PCA, no sample was removed based on ethnicity. A second PCA was performed to compute eigenvectors to include in the association analysis. We conducted a linear regression analysis to test for CD40 protein level-SNP association (PLINK). Ten eigenvectors were included as covariates in the linear model.

Details of the eQTL analysis have been previously described [30]. In short, we assessed the effect of rs4810485 on CD40 in whole peripheral blood in a collection of 1,469 samples (1,240 samples run on the Illumina HT12v3 platform, 229 samples run on the Illumina H8v2 platform). We used a Spearman rank correlation and meta-analysis using a weighted Z-method to calculate statistical significance of the rs4810485 G alleles CD40 gene expression levels.

### BL2 cells and shRNA targeting of CD40

BL2 cells were purchased from DSMZ (Germany; Cat.# ACC 625). Both BL2/shRNA cell line and BL2-NFκB-Luc cell line were derived from BL2 cells, as described below. All cells were cultured in RPMI 1640 medium (Life Technologies, Inc) supplemented with 10% FBS. For CD40 activation, cells were incubated at 37°C for 15 minutes with 64 ng/ml trimerized CD40 ligand (tCD40L). Two independent BL2 cell lines were generated, in which *CD40* was knocked-down using a HuSH shRNA Plasmid, pGFP-V-RS (Origene; Cat.# TR30007). A double-stranded DNA oligo containing a hairpin structure with sequence specific to human CD40 gene (sequence below) [33] was cloned into the pGFP-V-RS, according to manufacture specifications (Origene).

5′GATCGGCGAATTCCTAGACACCTGTTTCAAGAGAACAGGTGTCTAGGAATTCGCTTTTTTGAAGCT3′

The plasmid (20 µg) was linearized by ScaI and cotransfected into BL2 cells with a puromycin selection vector by electroporation at 300 V and 950 µF. Transfected cells were cultured in regular medium for 2 hours before they were serially diluted and plated into 96-well plates. For selection, cells were grown in 0.3 µg/ml puromycin. Single colonies were picked two weeks later and CD40 levels were measured by Western blot and FACS analysis.

### Analysis of p65 phosphorylation

For the immunoblot detection, 0.25×10^6^ BL2 cells were activated either with or without tCD40L. Whole-cell lysate was prepared with 10 µl RIPA buffer and then subjected to electrophoresis on a 7.5% SDS-PAGE gel under reducing conditions. Proteins were electro-blotted onto a nitrocellulose membrane. The membranes were detected by antibodies for CD40 (Santa Cruz; cat#: sc-13128), NF-κB p65 (Cell signaling; cat#: 4767) and phospho-NF-κB p65 (Ser536) (Cell signaling; cat#: 3033).

### Luciferase assay to detect the activity of NF-κB in BL2 and Ramos cells

A cignal lenti luciferase reporter construct driven by a basal promoter element (TATA box) joined to tandem repeats of the NF-κB response element was infected into BL2 and Ramos RA-1 cells, according to manufacture specifications (Qiagen; Cat.# CLS-013L). We call these two lines as BL2-NFκB-luc and Ramos-NFκB-luc, respectively. Single colonies were selected on 96 well plates with 0.3 µg/ml puromycin. Positive clones were further screened by luciferase assay with Steady-Glo assay system (Promega) after 4 hr activation by 64 ng/ml tCD40L. Since the expression of luciferase gene is controlled by the activity of NF-κB, we were able to measure the activity of NF-κB following activation with tCD40L by measuring the activity of luciferase. For LPS activation in BL2 cells, a BL2-NFκB-luc line was transfected with episomal DNA of TLR4 (pUNO1-hTLR4a, InvivoGen) for high levels of TLR4 expression. We call this line as BL2-TLR4-NFκB-luc.

### High-throughput screen for inhibitors and activators for CD40 signaling pathway

The high-throughput screening assay was optimized in 384-well format in collaboration with the Broad Institute Probe Development Center (BIPDeC). Briefly, 10 µL BL2-NFκB-luc cells at 25K cells/well were plated into each well on a 384-well plate (Perkin Elmer; Cat.# 6008230) using a Multidrop Combi dispenser (Thermo). For each compound, DMSO or positive control IKK inhibitor VII, 25 nL was transferred using a pin tool (Cybio). The final concentration for each compound was 9.4 µM; DMSO 0.25%; and IKK inhibitor VII 50 µM. After cells were incubated at 37°C for 1 hour, 10 uL (192 ng/mL) tCD40L was added to make a final concentration of 64 ng/ml. Cells were incubated at 37°C for 4.5 hrs before 5 uL 1X Steady-Glo luciferase substrate was added. Luciferase activity was read after 5 min using LJL analyst plate reader (LJL BioSystems). Each compound was tested in duplicate. A complete list of the compounds can be found in Table S3. We refer to this set of compounds as our “2K screening” set.

Seventy-three of 81 “hit” compounds from the primary screen were advanced into a series of counter-screens. The 73 compounds were selected because stock concentrations of each compound were available for dose-titration experiments. Each compound was tested across a range of concentrations from 50 µM to 0.39 µM (2-fold decrease between doses). In addition to screening BL2-NFκB-luc cells activated with tCD40L, we also screened BL2-TLR4-NFκB-luc cells activated with 16 ng/ml LPS (Sigma). We screened an additional B cell line transfected with the same luciferase reporter contruct, Ramos-NFκB-luc cells, and activated with 64 ng/ml tCD40L and 64 ng/ml TNFα (eBioscience). Cell viability of both lines was evaluated by adding 5 ul 0.5X CellTiter-glo.

### Measurement of CD86 expression in human CD19+ primary B cells

CD86 protein levels were measured in human primary CD19+ B cells from the PhenoGenetic Project purified by MACs (Milteyi Biotec; Cat# 130-091-151). Purified human primary CD19+ B cells (1×10^6^) were pre-incubated with 10 ng/ml IL4 and different concentrations of drugs in each well of a 24-well plate at 37°C for one hour. Cells were activated with and without 64 ng/ml tCD40L. After 48 hours, cells were stained with anti-CD19-FITC/anti-CD86-PE (Biolengd; Cat#305405). CD86 expression was measured by PE GeoMFI on CD19+ gated B cells.

### Statistical analysis of compound screening data

The raw signals of each 384-well microtiter wells were normalized using the “Neutral Controls minus Inhibitors” method in Genedata Assay Analyzer (v7.0.3). The median raw signal of the intraplate neutral-control wells was set to a normalized activity value of 0. The median raw signal of the intraplate positive-control wells was set to a normalized absolute activity value of 100. The plate pattern correction algorithm “Assay Median” in Genedata (v7.0.3) was applied to the normalized plate data. We used DMSO neutral controls to define 95% confidence intervals (CI) of our 2K screening compounds. We defined “hits” as those compounds outside of 95% CI in both dimensions of the replicate experiments. This led to 86 compounds that inhibited luciferase activity, consistent with our positive IKK inhibitor control. We defined a compound as promiscuous if it satisfies both of the following two rules: (1) the number of assays in which it has been tested is larger than 50, and (2) the ratio between the number of hits and the number of assays in which it has been screened is larger than 0.25. Based on these criteria, we found 40 promiscuous compounds, 5 of which had >2SD inhibition of luciferase activity, yielding 81 compounds that inhibited luciferase activity. A list of all compounds tested, including the annotation of 40 promiscuous compounds, can be found in Tables S3 and S4.

To calculate relative IC_50_, the *nls* function in the R package of stats (2.14 version) was used to fit the four-parameter logistic non-linear regression model as follows. This method represents the nonlinear (weighted) least-squares estimates of the parameters of a nonlinear model, with the equation (x_n_ is concentration and y_n_ is luciferase intensity): 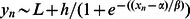. It estimates the four parameters *L*, *h*, α, and β. The starting point of *L* is the maximum value at smallest concentration; the starting point of *h* is the minimum difference between the minimum value at the largest concentration and the average value at smallest concentration. The starting point of α and β are 4 and 2, respectively. The R function smooth.spline was used to smooth the estimated points in the curves. In some cases where a singular gradient happens, the parameters are not estimable; when a plateau was not observed, the curve was smoothed by loess.smooth function in R and the percentage was calculated by the observed values. The 95% confidence interval (CI) was computed based on the observed data and transferred into percentage. To calculate percent maximum inhibition for each compound, we determined the difference between the average activity at the lowest drug concentration and the average activity at the highest drug concentration. The percent maximum inhibition was then obtained by dividing the difference by the average activity at the lowest drug concentration.

## Supporting Information

Figure S1 Regional association plot of CD40 locus, following conditional analysis of rs4810485 in (A) case-control study of RA risk, and (B) CD40 protein levels; (C) Manhattan plot of ∼140,000 iChip SNPs tested for association with CD40 protein levels.
(DOCX)

Figure S2 Correlation between CD40 protein levels measured by flow cytometry in the same individuals at two different points in time (>3 months apart).
(DOCX)

Figure S3 BL2 cells were incubated with different concentration of IKK for 1 hr and then activated with 16 ng/ml tCD40L for 15 min. Western blot was probed by anti-phospho-p65, anti-p65 and anti-parp antibodies separately.
(DOCX)

Figure S4 Coverage of *CD40* exons by our pooled sequencing strategy.
(DOCX)

Figure S5 (A) Schematic of compound set enrichment analysis: (i) we used text-mining to annotate each compound with a “Pharmacological Action” MeSH term in PubChem; each Pharmacological Action term set contains multiple compounds; (ii) for each compound, we determine luciferase activity relative to the overall distribution: increased (red), neutral (white), or decreased (blue), where the white areas are defined by corrected values between −1 and 1. (iii) we test sets of compounds, annotated by Pharmacological Action terms, for enrichment of the distribution of compounds within each term set relative to the entire distribution of results. (B) The distribution of enrichment scores for 117 groups of pharmacological action terms with ≥3 compounds. Red indicates positive enrichment and blue indicates negative enrichment relative to all sets tested. The dashed line delineates threshold of statistical significance, given the number of independent hypotheses (i.e., compound sets) tested. The 5 compound sets that surpass this level of significance are labeled. (C) The results of top 5 compound sets from the enrichment analysis. Each strip shows the distribution of all results, with black lines indicating specific compounds within each set. The top 5 compound sets are shown, with the number of compounds that inhibit luciferase activity, total number of compounds with the specific Pharmacological Action term annotation, and P-value for enrichment shown at the top (from the Wilcoxon mean rank test).
(DOCX)

Figure S6 Corticosteroid compounds and chemical structure. (A) Core structures of the corticosteroid hits. Chemical similarity of 28 corticosteroids and their analogs. Since there are only 45 corticosteroids among 1,982 compounds tested, this finding represents a significant enrichment among hits (*P*<10^−16^). (B) Functional groups of the corticosteroid hits that create rings.
(DOCX)

Figure S7 Dose-response curves for two “known” and “unknown” compounds in Ramos-NFkB-Luc cells.
(DOCX)

Table S1 Description of samples used in immunochip (iChip) association study of case-control status.
(DOCX)

Table S2 (A) List of each equivalent SNP; r2 with rs4810485; (B) missense SNPs discovered by sequencing, with number of counts in cases and controls.
(DOCX)

Table S3 (A) Complete list of chemical compounds use in 2K pilot screen; (B) top 81 hits.
(XLSX)

Table S4 List of promiscuous chemical compounds from 2K pilot screen.
(XLSX)

Table S5 Corticosteroid compounds and chemical structure.
(DOCX)

Table S6 Percent inhibition of luciferase activity or cell viability at 50 uM compound.
(DOCX)

Text S1 A description of additional methods is provided. This includes details on CD40 sequencing and compound set enrichment analysis.
(DOCX)
