# Supplementary figures and images for: Human Genetics in Rheumatoid Arthritis Guides a High-Throughput Drug Screen of the CD40 Signaling Pathway
Source: PLoS Genet. 2013 May 16;9(5):e1003487. doi: 10.1371/journal.pgen.1003487 (PMC3656093; doi:10.1371/journal.pgen.1003487)

A.

B.


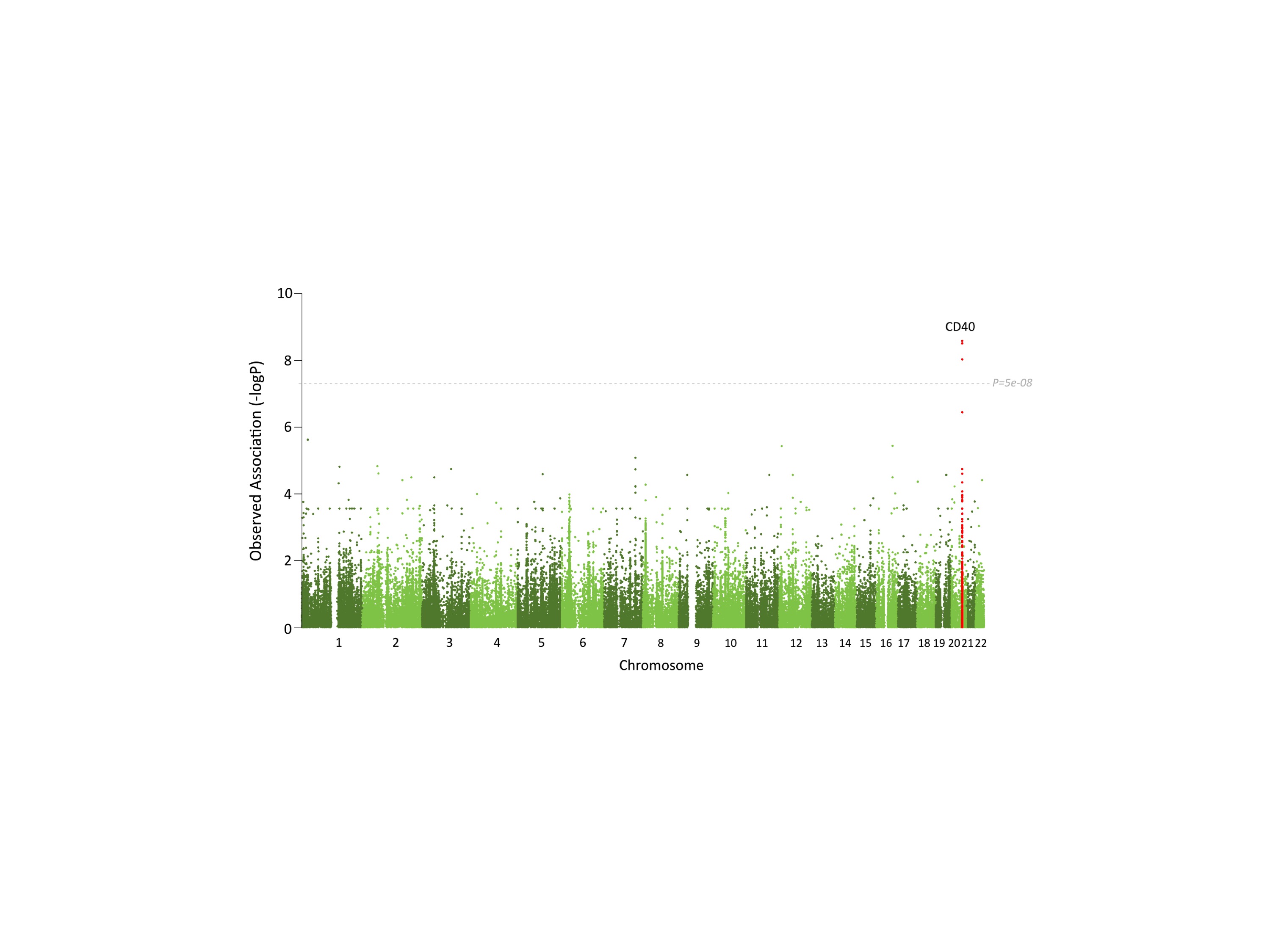
C.

Supplement: Figure S1 — Regional association plot of CD40 locus, following conditional analysis of rs4810485 in (A) case-control study of RA risk, and (B) CD40 protein levels; (C) Manhattan plot of ∼140,000 iChip SNPs tested for association with CD40 protein levels. (DOCX) [file pgen.1003487.s001.docx]

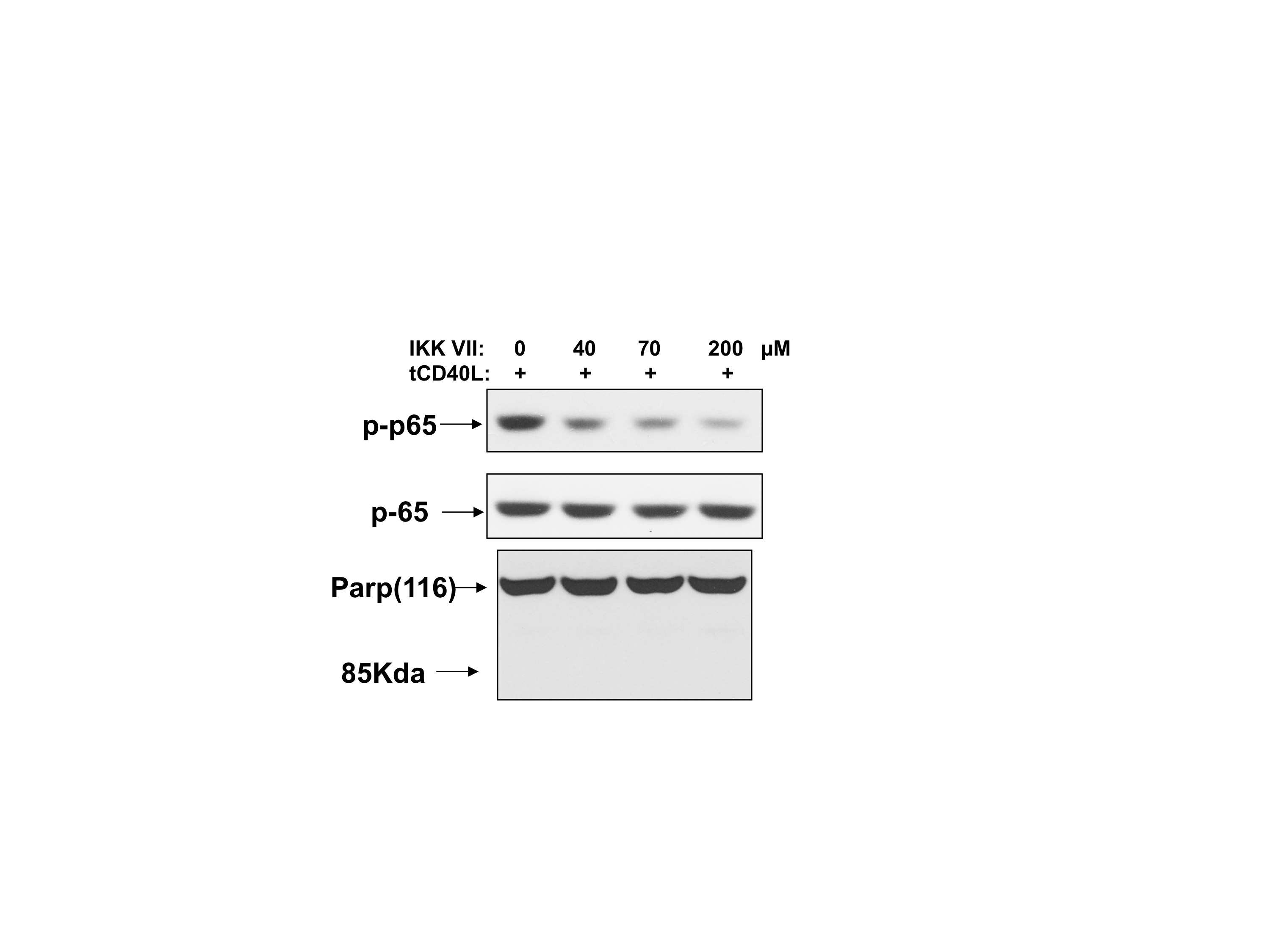

Supplement: Figure S3 — BL2 cells were incubated with different concentration of IKK for 1 hr and then activated with 16 ng/ml tCD40L for 15 min. Western blot was probed by anti-phospho-p65, anti-p65 and anti-parp antibodies separately. (DOCX) [file pgen.1003487.s003.docx]

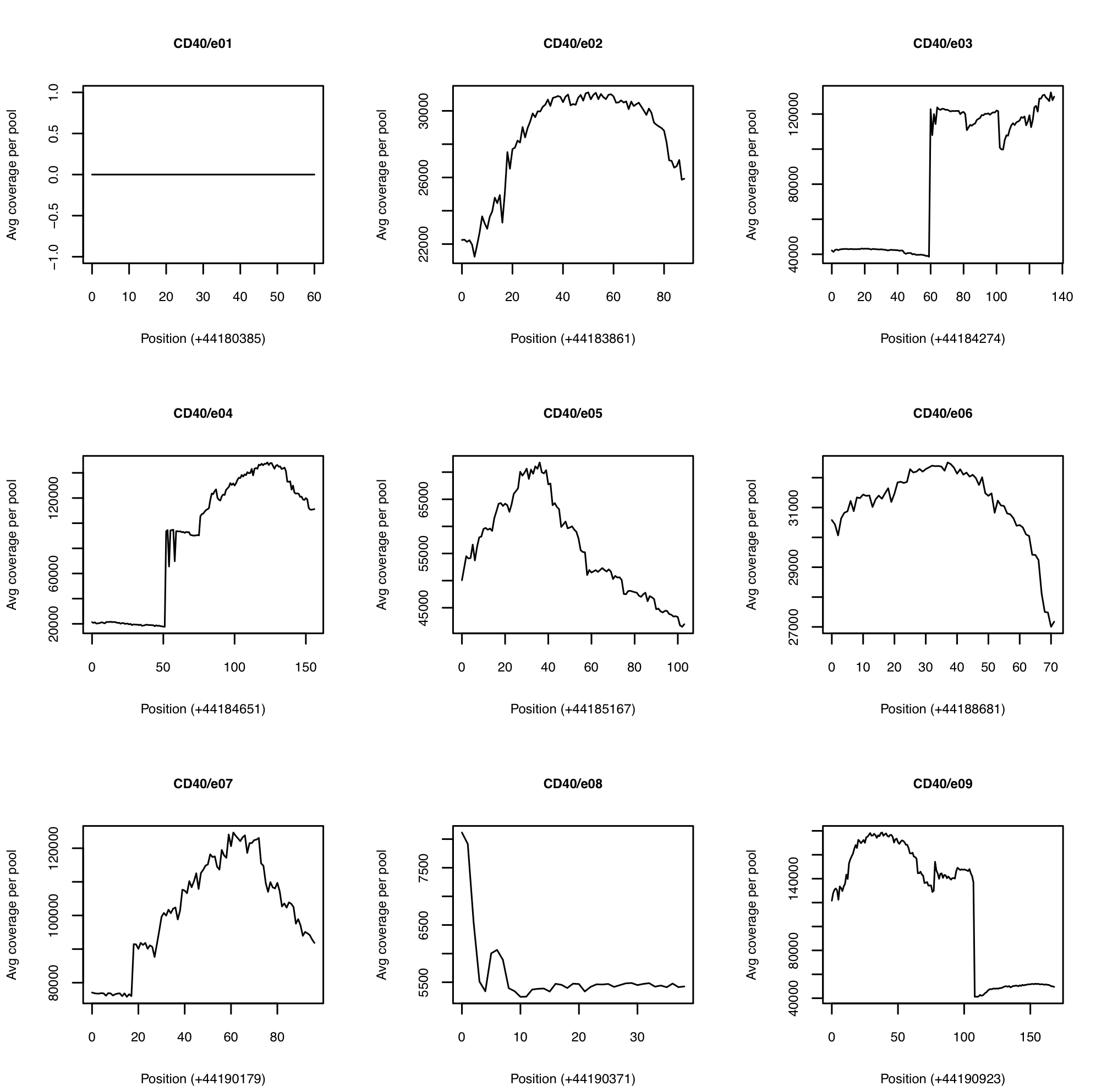

Supplement: Figure S4 — Coverage of CD40 exons by our pooled sequencing strategy. (DOCX) [file pgen.1003487.s004.docx]

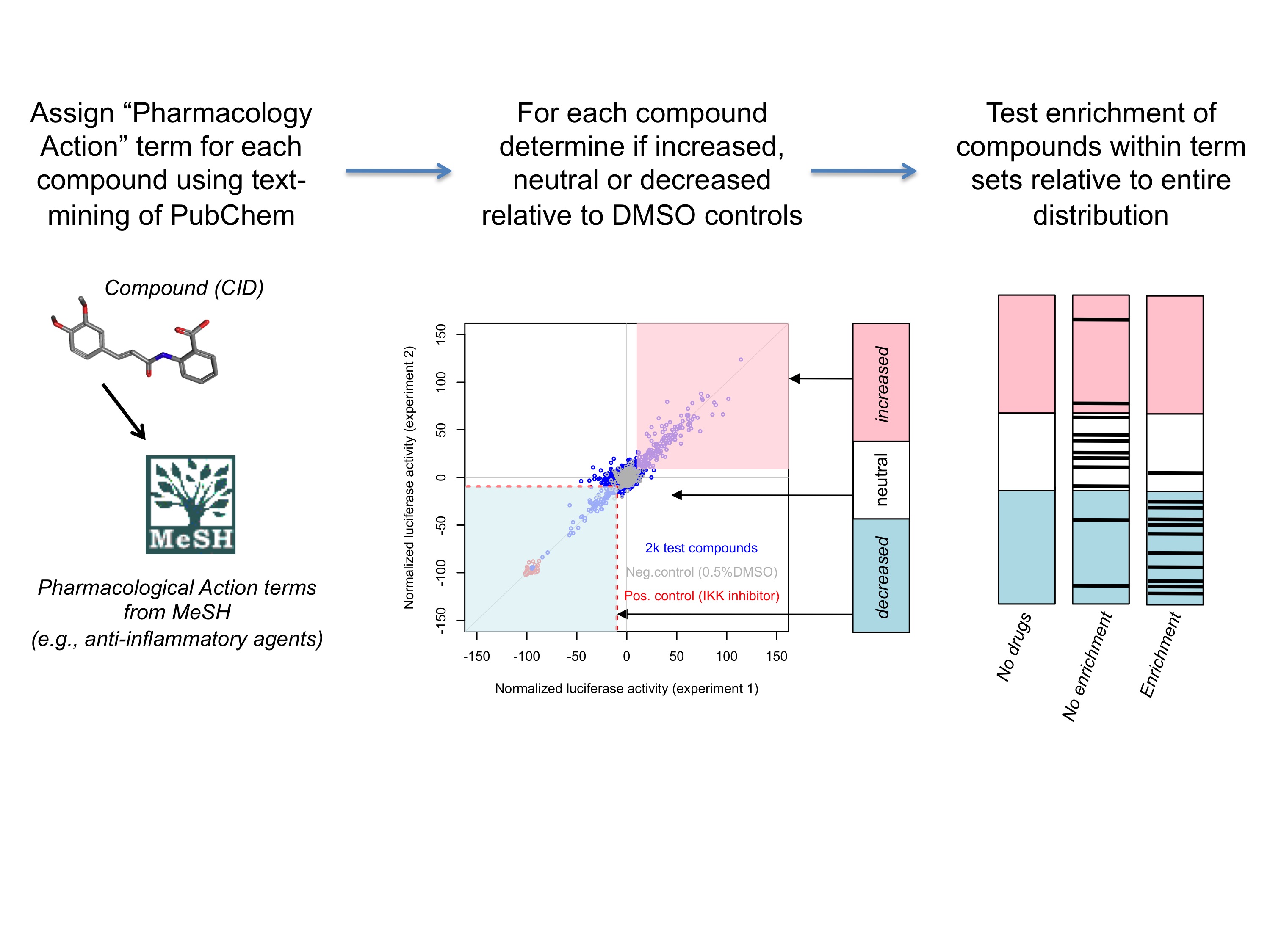
A.


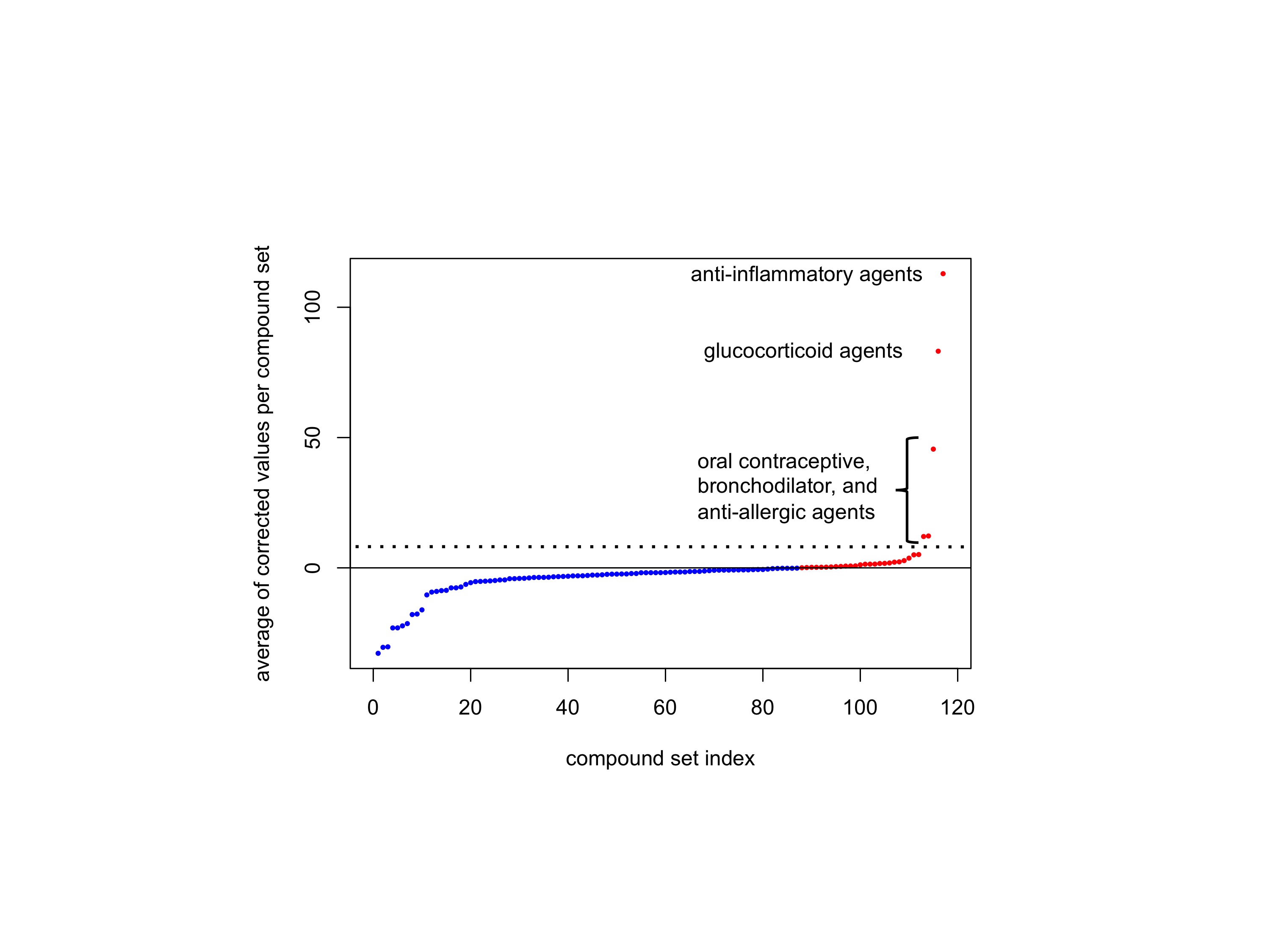
B.


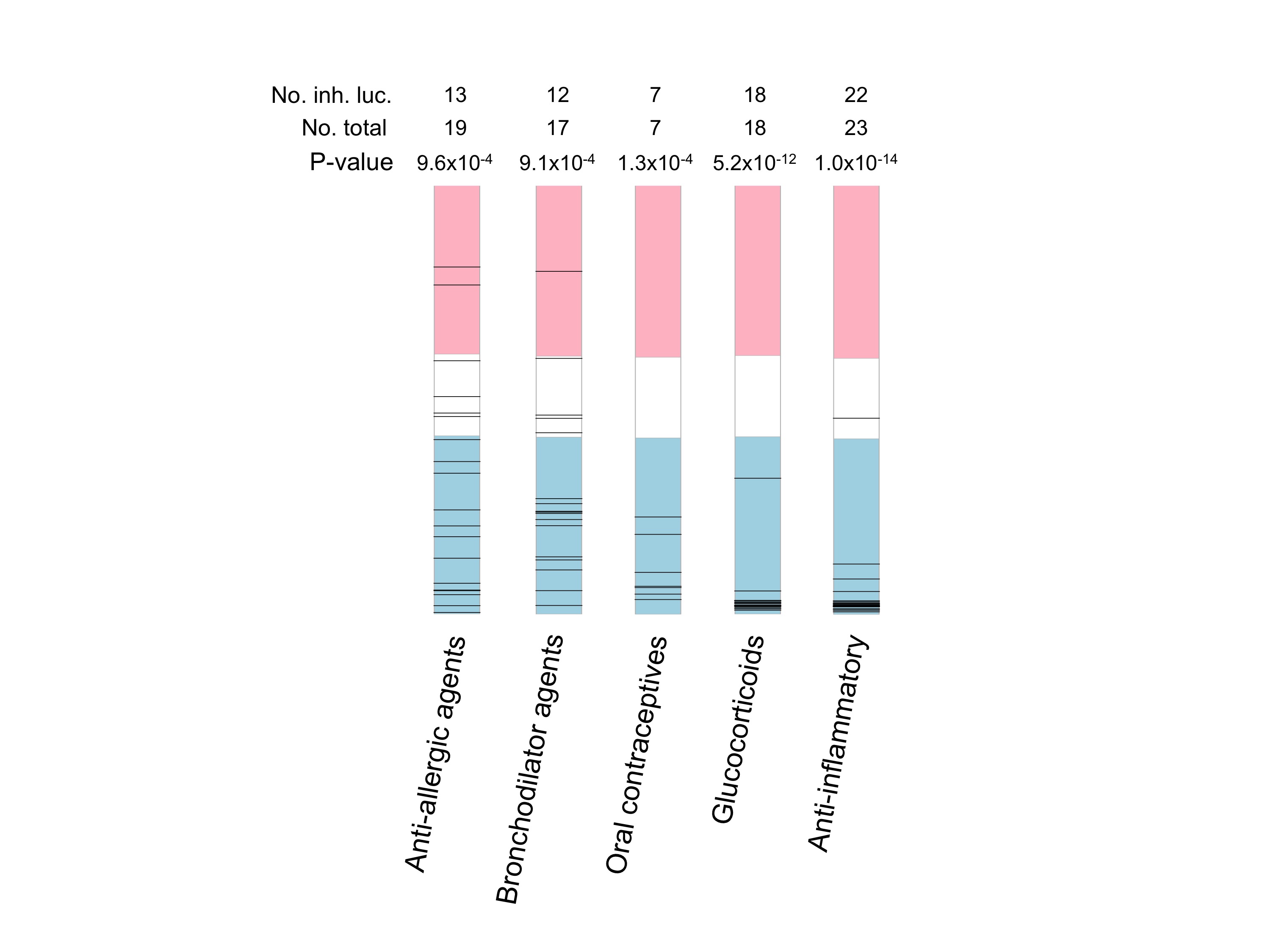
C.

Supplement: Figure S5 — (A) Schematic of compound set enrichment analysis: (i) we used text-mining to annotate each compound with a “Pharmacological Action” MeSH term in PubChem; each Pharmacological Action term set contains multiple compounds; (ii) for each compound, we determine luciferase activity relative to the overall distribution: increased (red), neutral (white), or decreased (blue), where the white areas are defined by corrected values between −1 and 1. (iii) we test sets of compounds, annotated by Pharmacological Action terms, for enrichment of the distribution of compounds within each term set relative to the entire distribution of results. (B) The distribution of enrichment scores for 117 groups of pharmacological action terms with ≥3 compounds. Red indicates positive enrichment and blue indicates negative enrichment relative to all sets tested. The dashed line delineates threshold of statistical significance, given the number of independent hypotheses (i.e., compound sets) tested. The 5 compound sets that surpass this level of significance are labeled. (C) The results of top 5 compound sets from the enrichment analysis. Each strip shows the distribution of all results, with black lines indicating specific compounds within each set. The top 5 compound sets are shown, with the number of compounds that inhibit luciferase activity, total number of compounds with the specific Pharmacological Action term annotation, and P-value for enrichment shown at the top (from the Wilcoxon mean rank test). (DOCX) [file pgen.1003487.s005.docx]

Table S6: Percent decrease in luciferase activity or cell viability at 50 uM compound


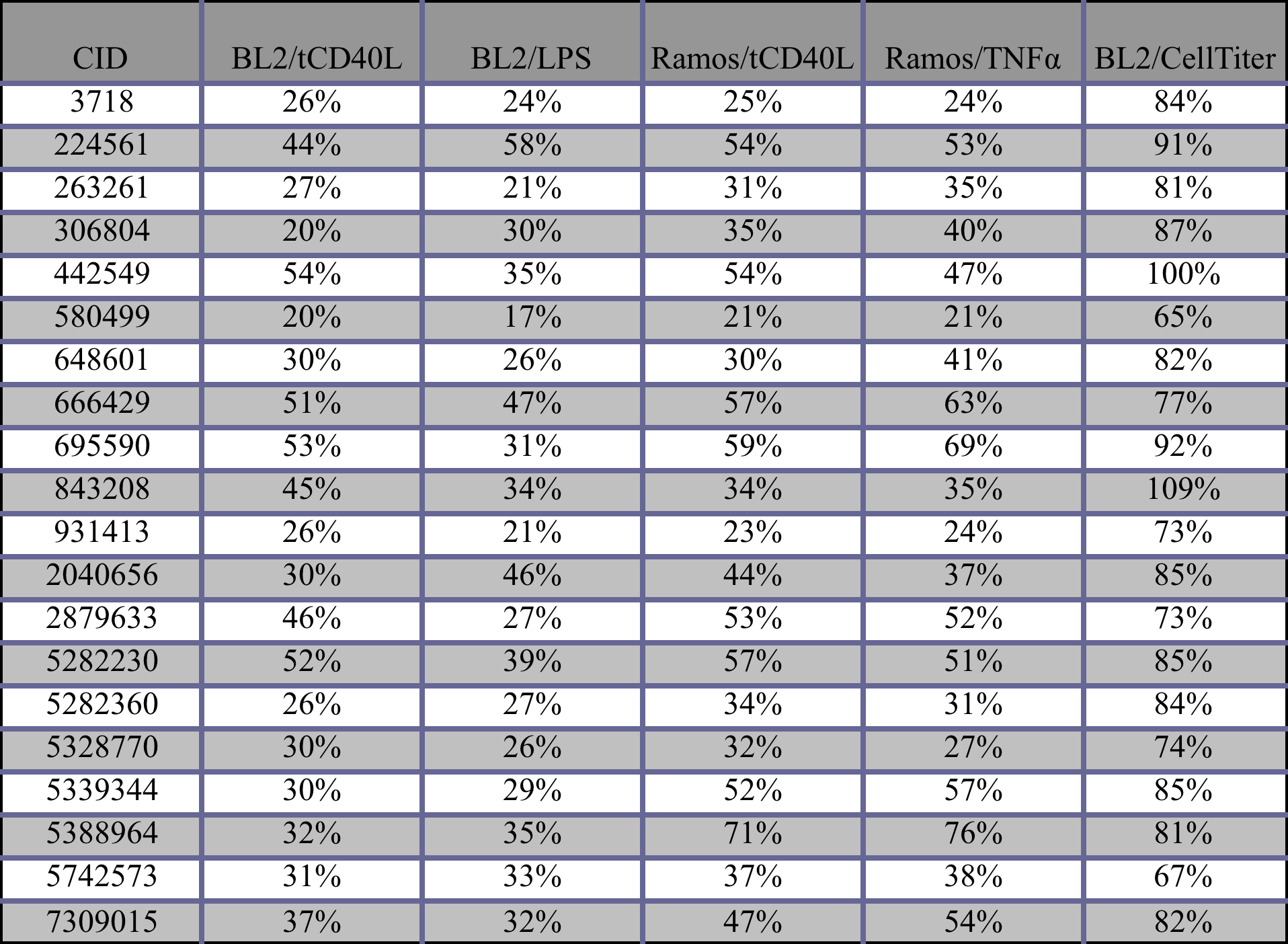

Supplement: Table S6 — Percent inhibition of luciferase activity or cell viability at 50 uM compound. (DOCX) [file pgen.1003487.s013.docx]
