# Supplementary material for: Human Genetics in Rheumatoid Arthritis Guides a High-Throughput Drug Screen of the CD40 Signaling Pathway
Source: PLoS Genet. 2013 May 16;9(5):e1003487. doi: 10.1371/journal.pgen.1003487 (PMC3656093; doi:10.1371/journal.pgen.1003487)
Supplement: Figure S6 — Corticosteroid compounds and chemical structure. (A) Core structures of the corticosteroid hits. Chemical similarity of 28 corticosteroids and their analogs. Since there are only 45 corticosteroids among 1,982 compounds tested, this finding represents a significant enrichment among hits (P<10−16). (B) Functional groups of the corticosteroid hits that create rings. (DOCX) [file pgen.1003487.s006.docx]

A.

Core 1

Core 2

B.

Functional group 1 Functional group 2 Functional group 3
