# Supplementary material for: Human Genetics in Rheumatoid Arthritis Guides a High-Throughput Drug Screen of the CD40 Signaling Pathway
Source: PLoS Genet. 2013 May 16;9(5):e1003487. doi: 10.1371/journal.pgen.1003487 (PMC3656093; doi:10.1371/journal.pgen.1003487)
Supplement: Table S1 — Description of samples used in immunochip (iChip) association study of case-control status. (DOCX) [file pgen.1003487.s008.docx]

| **Collection** | **Geographic origin** | **Cases** | **Controls** | **Case antibody status** | **Genotyping platform** |
| --- | --- | --- | --- | --- | --- |
| UK | United Kingdom | 2406 | 8430 | 100% anti-CCP+ | Illumina Immunochip |
| SE- EIRA | Sweden | 1762 | 1940 | 100% anti-CCP+ | Illumina Immunochip |
| US | USA | 1803 | 2134 | 100% anti-CCP+ | Illumina Immunochip |
| Dutch | The Netherlands | 330 | 2004 | 100% anti-CCP+ | Illumina Immunochip |
| SE-Umea | Sweden | 524 | 963 | 100% anti-CCP+ | Illumina Immunochip |
| ES | Spain | 397 | 399 | 100% anti-CCP+ | Illumina Immunochip |
