# Supplementary material for: Human Genetics in Rheumatoid Arthritis Guides a High-Throughput Drug Screen of the CD40 Signaling Pathway
Source: PLoS Genet. 2013 May 16;9(5):e1003487. doi: 10.1371/journal.pgen.1003487 (PMC3656093; doi:10.1371/journal.pgen.1003487)
Supplement: Table S2 — (A) List of each equivalent SNP; r2 with rs4810485; (B) missense SNPs discovered by sequencing, with number of counts in cases and controls. (DOCX) [file pgen.1003487.s009.docx]

|  |  |  |  |  | RA risk allele |  | RA case-control association meta-analysis | | |  | CD40 protein level association | |
| --- | --- | --- | --- | --- | --- | --- | --- | --- | --- | --- | --- | --- |
| SNP | Coordinates*^a^* | R^2^*^b^* | Annotation | Alleles |  | MAF | OR (95% CI)*^c^* | nb*^d^* | P |  | BETA*^e^* | P |
| rs4810485 | chr20:44181354 | 1 | CD40 intron1 | T/G | G | 0.24 | 1.17 (1.11-1.23) | 6 | 1.44e-09 |  | 61.28 | 3.08e-09 |
| rs1883832 | chr20:44180389 | 0.998 | CD40 utr-5 | T/C | C | 0.24 | 1.13 (1.07-1.2) | 5 | 1.31e-05 |  | 60.29 | 9.28e-09 |
| rs6032662 | chr20:44167717 | 0.983 | intergenic | C/T | T | 0.24 | 1.17 (1.11-1.23) | 6 | 1.36e-09 |  | 60.29 | 9.28e-09 |
| rs6074022 | chr20:44173603 | 0.982 | intergenic | C/T | T | 0.24 | 1.17 (1.11-1.23) | 6 | 2.32e-09 |  | 60.29 | 9.28e-09 |
| rs6032664 | chr20:44172826 | 0.982 | intergenic | A/T | T | 0.24 | 1.17 (1.11-1.23) | 6 | 2.41e-09 |  | 60.29 | 9.28e-09 |
| rs6065926 | chr20:44169261 | 0.980 | intergenic | A/G | C | 0.24 | 1.17 (1.11-1.23) | 6 | 2.46e-09 |  | 58.91 | 3.56e-07 |
| rs4239702 | chr20:44182658 | 0.860 | CD40 intron 1 | T/C | C | 0.27 | 1.15 (1.09-1.21) | 6 | 1.75e-08 |  | 61.79 | 2.58e-09 |

*^a^ Coordinates in hg18*

*^b^ Linkage desequilibrium relative to rs4810485 in Immunochip*

*^c,e^ Odds ratio and BETA of RA risk allele*

*^d^ Number of collections meta-analysed*

| **chr:position** | **Type** | **Alleles** | **rs_ID** | **Amino acid change** | **Counts** | |
| --- | --- | --- | --- | --- | --- | --- |
|  |  |  |  |  | **cases** | **controls** |
| chr20:44183929 | missense | T/C | - | S115P | 0 | 1 |
| chr20:44190969 | coding-synon | C/T | - | P715P | 0 | 2 |
| chr20:44190931 | missense | C/G | rs11086998 | P679A | 4 | 5 |
