# Supplementary material for: Human Genetics in Rheumatoid Arthritis Guides a High-Throughput Drug Screen of the CD40 Signaling Pathway
Source: PLoS Genet. 2013 May 16;9(5):e1003487. doi: 10.1371/journal.pgen.1003487 (PMC3656093; doi:10.1371/journal.pgen.1003487)
Supplement: Table S5 — Corticosteroid compounds and chemical structure. (DOCX) [file pgen.1003487.s012.docx]

Table S5: corticosteroid compounds and chemical structure; please see Figure S6 for chemical structure Core structure and functional groups 1-3.
