# Supplementary material for: Human Genetics in Rheumatoid Arthritis Guides a High-Throughput Drug Screen of the CD40 Signaling Pathway
Source: PLoS Genet. 2013 May 16;9(5):e1003487. doi: 10.1371/journal.pgen.1003487 (PMC3656093; doi:10.1371/journal.pgen.1003487)
Supplement: Text S1 — A description of additional methods is provided. This includes details on CD40 sequencing and compound set enrichment analysis. (DOCX) [file pgen.1003487.s014.docx]

**SUPPLEMENTARY NOTE ON METHODS**

*Sequencing the* CD40 *risk locus*

Sequence data at the *CD40* locus was generated as part of a larger experiment to perform pooled sequencing of 25 RA risk genes. Briefly, we combined DNA into 10 pools from CCP+ RA cases (n=500 total RA cases) and 13 pools from matched controls (n=650 total controls), with each pool containing an equal amount of DNA from 50 individuals. All cases were matched to controls using principal components analysis from either GWAS data or ancestry informative markers ^1-3^. For each pool, we performed PCR amplification to capture the target sequence, which included 247 exons (37.8kb); each amplicon was approximately 125 bp. PCR successfully captured 92% of targeted bases (34.6 kb). All PCR amplicons were combined in equimolar amounts and paired-end sequencing performed at the Broad Institute using Illumina Genome Analyzer II. Data were visualize and analyzed using the Genome Analysis Tool Kit (GATK) ^4^. Given the pooled nature of the data, variant calling was implemented in Syzygy, similar to that performed in other pooled sequencing studies from the Broad Institute ^5^ We applied several filters (in the following order) on the variants to identify a set of high-confidence SNPs (n=320 SNPs in total). (1) We required that each variant detected have 2000-fold coverage on either the forward or reverse strands with a total of 5000-fold coverage in total per base, resulting in a minimum 20-fold coverage per chromosome within a pool. (2) We required concordant allele frequencies on the positive and negative strands. (3) We considered the non-randomness of the noise spectrum of technical artifacts likely occurring from biased preference/cross-talk for different base signal channels. Syzygy has a trained algorithm to filter out SNPs that show evidence of this artifact and allows the user to filter out these variants. (4) To eliminate errors due to alignment and sequencing errors that manifest as runs of missense mutations, we filtered out all SNPs that clustered together within a 5 bp window centered on the SNP. (5) Because our 23 lanes of sequencing were run in 3 separate batches, we performed regression analysis to determine whether significant batch effects exist in our data. Following our stringent filtering criteria, 320 variants remained. Of these, 81 (or ~25%) were present in either dbSNP or the 1000 Genomes Project dataset, indicating that most of our SNPs were novel. Most of the 348 SNPs were non-synonymous SNPs (n=186; 4 nonsense, 182 misssense), with the remaining being either synonymous SNPs (n=121) or non-coding SNPs (n=13). PolyPhen-2 predicted that 80 out of the 182 missense SNPs were “damaging”. The Ti/Tv ratio of these variants was 3.64 (251 transitions, 69 transversions).

*Compound set enrichment tests*

To formally test if our hit compounds were functionally related, we tested for enrichment of “Pharmacological Action” MeSH terms in PubChem. We used text-mining ^6^ to assign a MeSH term(s) to each compound. Of the 1,982 compounds in our screen, 575 have at least one MeSH term. We tested for enrichment of our 81 hit compounds using two statistical methods, using the statistical program R: hypergeometric test for categorical data (81 hit compounds vs all other non-hit compounds; functions ‘dhyper’ and ‘phyper’) and the Wilcoxon mean rank gene set test (which uses the ranks of the corrected value for each compound in the 2K dataset instead of using a specific CI cutoff; implemented using the ‘geneSetTest’ function in the limma package). The enrichment *P*-value reported in the Results is from the hypergeometric test. These methods are similar to what has been described for gene set enrichment analysis of expression data ^7^, which others have also modified to facilitate interpretation of small molecule screens ^8^. A complete annotation file can be found in Supplementary Table 5.

We also tested for chemical similarity using the "Cluster Molecules" component in Pipeline Pilot (Accelrys, Inc.). We grouped small-molecule 'hits' according to their chemical similarity, and obtained one large cluster of 28 corticosteroids and their analogs (Supplementary Figure 6). Since there are only 45 corticosteroids among 1,982 compounds tested, this finding represents a significant enrichment among hits (*P*<10^-16^) according to hypergeometric test (implemented in R). The remaining significant results were all structural singletons with the exception of three pairs of similar compounds.

**REFERENCES**

1 Kurreeman, F. *et al.* Genetic Basis of Autoantibody Positive and Negative Rheumatoid Arthritis Risk in a Multi-ethnic Cohort Derived from Electronic Health Records. *Am J Hum Genet* **88**, 57-69, doi:S0002-9297(10)00643-9 [pii]

10.1016/j.ajhg.2010.12.007 (2011).

2 Price, A. L. *et al.* Discerning the ancestry of European Americans in genetic association studies. *PLoS Genet* **4**, e236 (2008).

3 Price, A. L. *et al.* Principal components analysis corrects for stratification in genome-wide association studies. *Nat Genet* **38**, 904-909 (2006).

4 McKenna, A. *et al.* The Genome Analysis Toolkit: a MapReduce framework for analyzing next-generation DNA sequencing data. *Genome Res* **20**, 1297-1303, doi:gr.107524.110 [pii]

10.1101/gr.107524.110 (2010).

5 Calvo, S. E. *et al.* High-throughput, pooled sequencing identifies mutations in NUBPL and FOXRED1 in human complex I deficiency. *Nat Genet* **42**, 851-858, doi:ng.659 [pii]

10.1038/ng.659 (2010).

6 Han, L., Suzek, T. O., Wang, Y. & Bryant, S. H. The Text-mining based PubChem Bioassay neighboring analysis. *BMC Bioinformatics* **11**, 549, doi:1471-2105-11-549 [pii]

10.1186/1471-2105-11-549 (2010).

7 Subramanian, A. *et al.* Gene set enrichment analysis: a knowledge-based approach for interpreting genome-wide expression profiles. *Proc Natl Acad Sci U S A* **102**, 15545-15550 (2005).

8 Shaw, S. Y. *et al.* Disease allele-dependent small-molecule sensitivities in blood cells from monogenic diabetes. *Proc Natl Acad Sci U S A* **108**, 492-497, doi:1016789108 [pii]

10.1073/pnas.1016789108 (2011).
