# Supplementary material for: Human Genetics in Rheumatoid Arthritis Guides a High-Throughput Drug Screen of the CD40 Signaling Pathway
Source: PLoS Genet. 2013 May 16;9(5):e1003487. doi: 10.1371/journal.pgen.1003487 (PMC3656093; doi:10.1371/journal.pgen.1003487)
Supplement: Supplementary file 9 [file pgen.1003487.s002.docx]

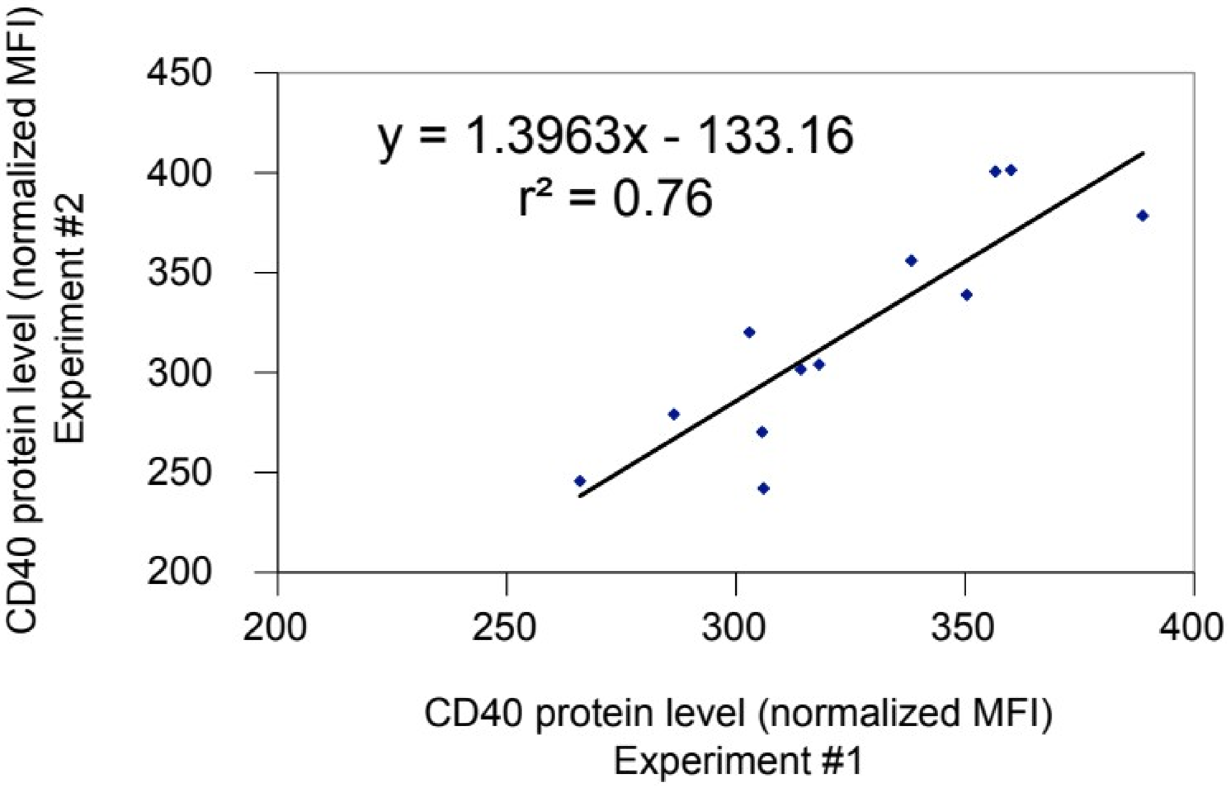

y = 1.3963x - 133.16
r² = 0.76
450
400
350
300
250
200
200
250
300
350
400
CD40 protein level (normalized MFI)
Experiment #1
CD40 protein level (normalized MFI)
Experiment #2
