# Supplementary material for: Human Genetics in Rheumatoid Arthritis Guides a High-Throughput Drug Screen of the CD40 Signaling Pathway
Source: PLoS Genet. 2013 May 16;9(5):e1003487. doi: 10.1371/journal.pgen.1003487 (PMC3656093; doi:10.1371/journal.pgen.1003487)
Supplement: Supplementary file 10 [file pgen.1003487.s007.docx]

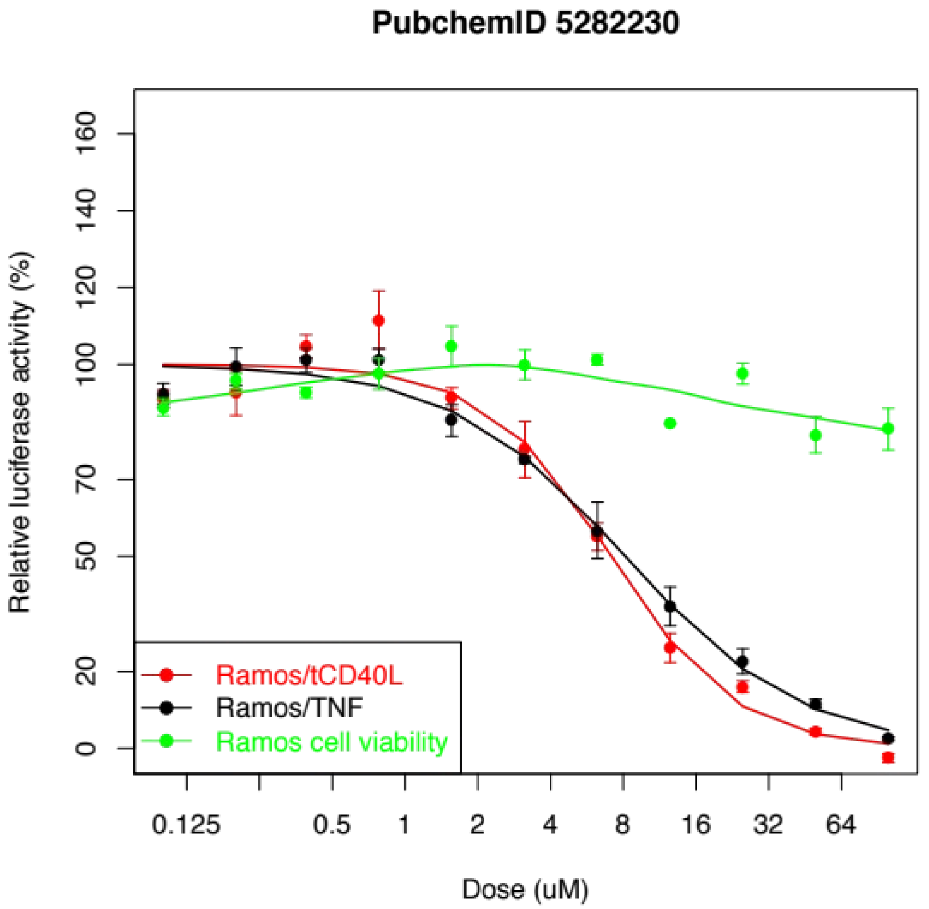
PubchemID 5282230
Relative luciferase activity (%)
0
20
50
70
100
120
140
160
0.125
0.5
1
2
4
8
16
32
64
Dose (uM)
Ramos/tCD40L
Ramos/TNF
Ramos cell viability

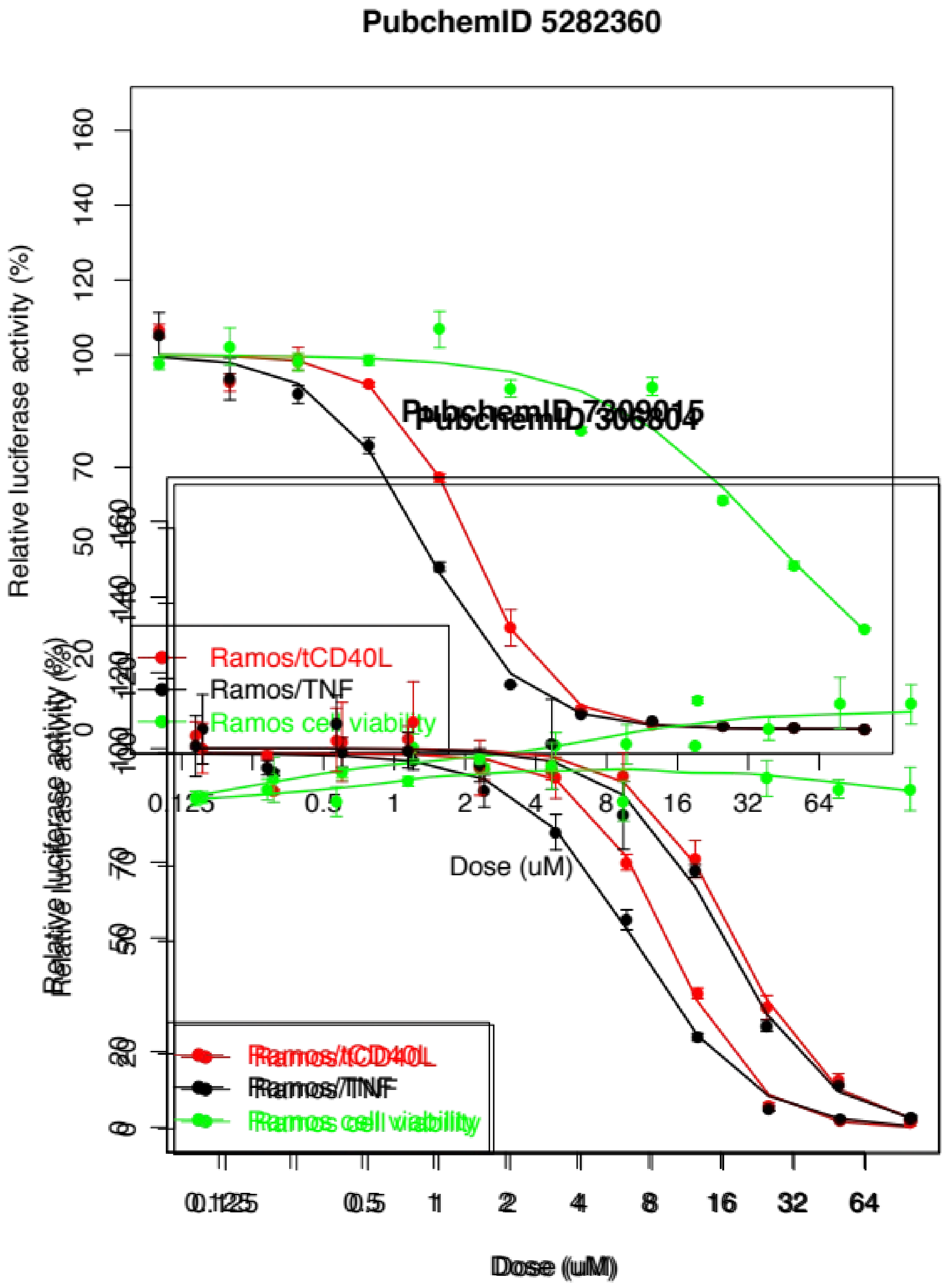
PubchemID 5282360
Relative luciferase activity (%)
Ramos/tCD40L
Ramos/TNF
Ramos cell viability
Dose (uM)
